# A Dislocation-Scale Characterization of the Evolution of Deformation Microstructures around Nanoindentation Imprints in a TiAl Alloy

**DOI:** 10.3390/ma11020305

**Published:** 2018-02-20

**Authors:** Antoine Guitton, Hana Kriaa, Emmanuel Bouzy, Julien Guyon, Nabila Maloufi

**Affiliations:** 1 Université de Lorraine, CNRS, Arts et Métiers ParisTech, LEM3, F-57000 Metz, France; hana.kriaa@univ-lorraine.fr (H.K.); emmanuel.bouzy@univ-lorraine.fr (E.B.); julien.guyon@univ-lorraine.fr (J.G.); nabila.maloufi@univ-lorraine.fr (N.M.); 2 Laboratory of Excellence on Design of Alloy Metals for low-mAss Structures (DAMAS)—Université de Lorraine, 57073 Metz, France

**Keywords:** TiAl alloys, dislocation, twinning, nanoindentation, ECCI

## Abstract

In this work, plastic deformation was locally introduced at room temperature by nanoindentation on a γ-TiAl-based alloy. Comprehensive analyses of microstructures were performed before and after deformation. In particular, the Burgers vectors, the line directions, and the mechanical twinning systems were studied via accurate electron channeling contrast imaging. Accommodation of the deformation are reported and a scenario is proposed. All features help to explain the poor ductility of the TiAl-based alloys at room temperature.

## 1. Introduction

Titanium aluminide alloys have attracted considerable attention due to their unique combination of properties, such as high specific strength and stiffness, good creep properties, and resistance against oxidation and corrosion [1,2], which make them suitable candidate materials for High-Temperature (HT) applications [3,4].

One of the main weaknesses of TiAl alloys is that they are brittle at Room Temperature (RT)—i.e., below their brittle-to-ductile transition temperature, which lies between 800 and 1000 °C [5]. Despite intense research on the HT behavior of TiAl alloys, literature suffers from a lack of understanding of their RT behavior, particularly regarding the elementary deformation mechanisms and the precise role of microstructures [6,7,8].

Among the several Ti-Al alloy phases, two of them are ordered at RT [4]: γ as the major phase and α_2_ as a minor phase. The α_2_ phase is hexagonal ($\frac{c}{a}=$ 0.8) with a DO19 structure, while the γ phase is tetragonal with a L1_0_ structure close to cubic ($\frac{c}{a}=\frac{c}{b}=$ 1.02). Therefore, six order variants are possible. They can be visualized as generated by a 120° rotation around the (1 1 1) plane normal [9].

The microstructures of γ-TiAl alloys are complex. A good compromise for balancing properties between RT plasticity, high strength, and good creep resistance at HT can be obtained for the duplex microstructure. It is constituted of a mixture of monolithic γ grains and small lamellar colonies of γ and α_2_ [10,11].

In dual-phase TiAl alloys, plastic deformation mainly occurs on the {1 1 1} planes of the γ phase by dislocation glide or twinning. It is strongly related to the ordered L1_0_ structure [12]: along the $\langle \bar{1}\;1\;0]$-directions, there is only one kind of atom (Ti or Al). In this case, dislocations are called ordinary dislocations, and their Burgers vectors are $\frac{1}{2}\langle 1\;1\;0]$ types. Because Ti-atoms and Al-atoms interchange in 〈0 1 1]-directions, the $\langle 1\;1\;\bar{2}]$ and the 〈1 0 1] dislocations are called superdislocations. These two types of superdislocations can undergo various dissociations into superpartials (i.e., partial dislocations with the associated planar faults). In addition, true twinning along $\frac{1}{6}\langle 1\;1\;\bar{2}]\{1\;1\;1\}$ occurs that does not alter the ordered L1_0_ structure of the γ-TiAl. Because of the specific structure of the γ-TiAl, it is relatively easy to know the direction for either slip of ordinary dislocations or for true twinning when the slip/twin plane is known [12]. Note also that at RT twinning and then glide of ordinary dislocations are the easiest deformation modes [2,7,8]. In this manner, Kauffmann et al. suggested that increasing deformation leads to the nucleation of only a few new mechanical twins, since the dislocation movement becomes more dominant with increasing strain [8].

Although it is accepted that the α_2_ phase does not contribute to the deformation [6,12], evidence of prismatic slip $\langle 1\;\bar{2}\;1\;0\rangle \left\{1\;0\;\bar{1}\;0\right\}$, basal slip $\langle 1\;\bar{2}\;1\;0\rangle \left(0\;0\;0\;1\right)$, and pyramidal slip $\langle 1\;1\;\bar{2}\;\bar{6}\rangle \left\{1\;\bar{2}\;1\;1\right\}$ was reported [12].

Among the difficulties encountered for understanding the mechanical behavior of TiAl-based alloys, most of our detailed knowledge of their deformation mechanisms has been deduced from Transmission Electron Microscopy (TEM) observations on an electron transparent lamella [7,13]. The investigation presented in this article focuses on the study of deformation mechanisms at the mesoscopic scale. With an original combination of experiments, we investigate the evolution of deformation microstructures at RT in the γ phase of a dual-phase bulk TiAl alloy. Because of the RT brittleness of this material, plastic deformation is induced by nanoindentation. The solid confinement around the indent maintains the integrity of the sample, while applying the load. Note also that nanoindentation is a surface technique, so the stress state at the specimen surface is different from that in the volume. The evolution of the microstructures was characterized by accurate Electron Channeling Contrast Imaging (aECCI) before and after deformation.

## 2. Materials and Methods

The fully dense Ti–46.8Al–1.7Cr–1.8Nb (at %) sample was obtained in the form of investment cast-bars (diameter 15 mm, height 230 mm) from Howmet. The as-received bars were hot isostatically pressed at 1250 °C and 125 MPa for 4 h, then subjected to a homogenization treatment in a furnace under vacuum at 1270 °C for 24 h [14]. Then, the sample was ground using silicon carbide paper and then polished with a 1 µm diamond suspension. Finally, in order to produce a very flat surface and avoid any work hardening due to conventional grinding, a chemo-mechanical polishing was performed using a colloidal silica suspension. 

Because deformation occurs mainly in the γ-phase [5], plastic deformation was locally introduced in the γ phase by nanoindentation using the Ultra Nanoindentation Tester from Anton Paar (Buchs, Switzerland), equipped with a Berkovich indenter. The indents were organized in a regular array of 500 µN indents. For easier recognition, it was surrounded by 20 mN indents at a distance of a few hundred µm.

Detailed characterizations of microstructures before and after deformation were performed by aECCI using a Zeiss Auriga Scanning Electron Microscope (SEM, Oberkochen, Germany) operating at 10 kV. aECCI is a non-destructive method offering the ability to provide—inside a SEM—TEM-like diffraction contrast imaging of sub-surface defects (at a depth of about one hundred of nanometers) on centimetric bulk specimens. Defects such as dislocations can be characterized by applying the TEM extinction criteria [15,16]. Because the yield of BackScattered Electrons (BSE) depends drastically on the orientation of the crystal relative to the incident electron beam (i.e., optic axis of the SEM), obtaining the crystallographic orientation of the grain of interest with an accuracy of 0.1° is a preliminary step to aECCI [16]. The precise orientation of the crystal in the SEM coordinate system is given through Selected Area Channeling Pattern (SACP). To overcome this challenge, rocking the incident electron beam at a pivot point on the surface of a given grain of the sample provides High-Resolution Selected Area Channeling patterns (HR-SACPs) [17]. HR-SACPs cover an angular range of 4.4° and reach an accuracy for the orientation better than 0.1° with a spatial resolution less than 500 nm. Because of this small angular range, in order to obtain the orientation of the grain of interest, the HR-SACP was superimposed on an Electron BackScattered Diffraction (EBSD) pattern simulated at 0° using “Esprit DynamicS” software from Bruker (Billerica, MA, USA). Note that the reason for using an EBSD pattern (acquired at 70°) simulated at 0° is that the specimen is initially placed at 0° for aECCI. 

EBSD experiments were carried out on a Zeiss Supra 40 SEM (Oberkochen, Germany) operating at 20 kV. In order to discriminate the different order variants of γ-TiAl, fine EBSD analyses were performed at a step of 75 nm with Channel 5 as the indexation software.

## 3. Results

### 3.1. Characterization of the Microstructure around the Regions of Interest

**Figure 1:**
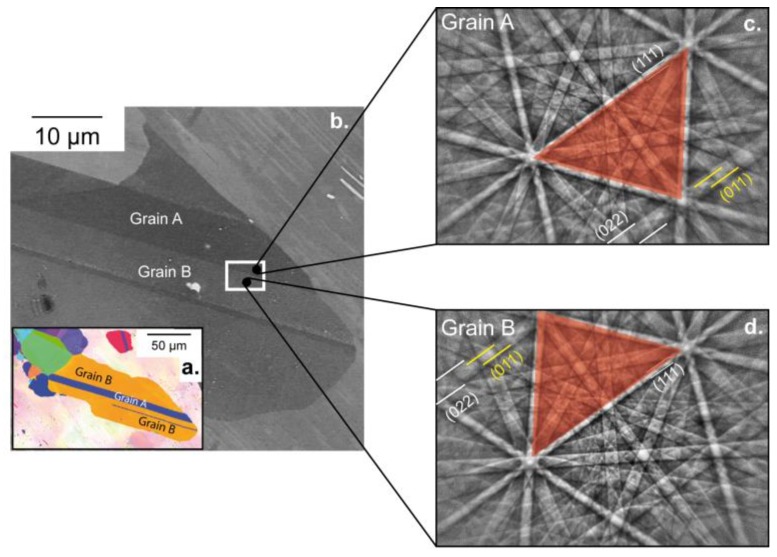
(**a**) Electron backscattered diffraction (EBSD) orientation map of the zone of interest. (**b**) Enhanced BSE image showing the microstructure before deformation. The nanoindentation array is localized in the white rectangle. (**c**,**d**) EBSD patterns corresponding to grains A and B.

**Figure 2:**
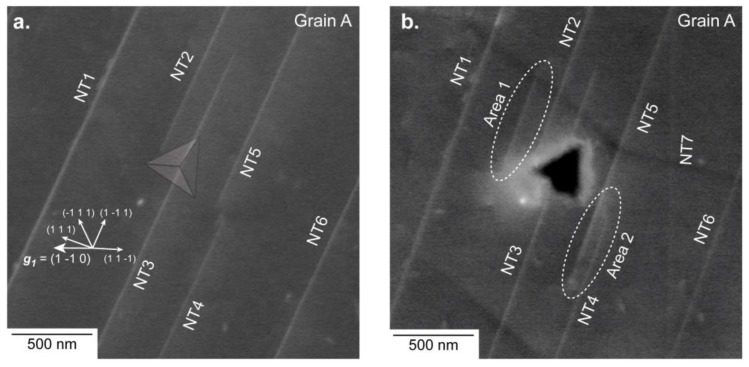
Region of interest 1 (ROI1) for which the surface is close to (4 5 7). (**a**) Accurate electron channeling contrast imaging (aECCI) obtained with $g_{1}=\;\left(1\;\bar{1}\;0\right)$ showing six $\left[1\;1\;\bar{2}\right]\left(1\;1\;1\right)$ Nano-Twins (NTs) and the position of the imprint (transparent Berkovich imprint). The white arrows indicate the trace of the {111} planes. (**b**) Enhanced BSE image showing the 500 µN indent. Two areas (labelled Areas 1 and 2) have changed. The NT7 slightly visible in (**b**) comes from a neighbor imprint.

**Figure 3:**
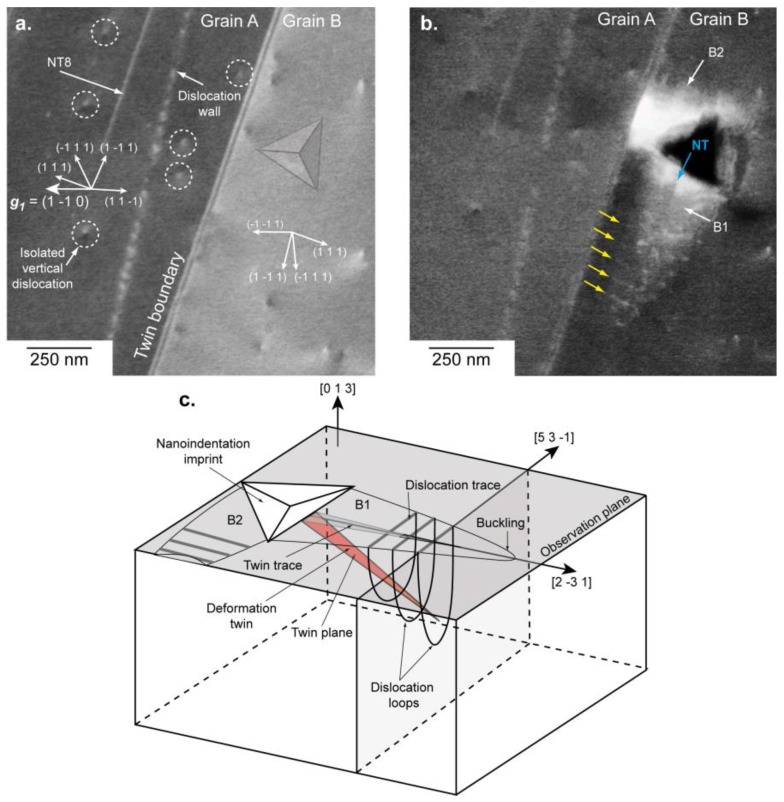
ROI2, where the surface plane is near (4 5 7) for twin A (left) and near (0 1 3) for grain B. The TB corresponds to $\left[1\;1\;\bar{2}\right]\left(1\;1\;1\right)$ the system. (**a**) aECCI obtained with $g_{1}=\;\left(1\;\bar{1}\;0\right)$ with the transparency position of the Berkovich imprint. The white arrows indicate the trace of the {1 1 1} planes. (**b**) Enhanced BSE image showing two buckling areas (labelled B1 and B2) clearly visible around the 500 µN indent. The blue arrow points to an NT and the yellow to dislocations. (**c**) 3D schematic of B1 and B2.

Figure 1a,b show the microstructure around the Regions Of Interest (ROIs): ROI1 on grain A away from any interfaces and ROI2 over both grains A and B. ROI1 and ROI2 are presented in Figure 2 and Figure 3, respectively. Note that References [18,19] mentioned that interfaces play an important role in TiAl alloys, thus controlling the yield stress.

Experimentally, the twin nature (true or pseudo-twin) was determined using high-resolution spot mode EBSD. Patterns were collected by manually pointing the electron beam at both sides of the Twin Boundary (TB). The corresponding EBSD patterns (Figure 1c,d) clearly indicate that grains A and B are true twin-related: for example, the red triangle formed by the three bands depicted in Figure 1c,d and the (0 1 1) superlattice band are in symmetrical position with respect to the unchanged (1 1 1) band when going from grain A to grain B.

The evolution of ROI1—before and after deformation—is presented in Figure 2a (ECC image) and Figure 2b (BSE micrograph). Due to a rapid contamination of the sample surface under the electron beam, controlling the channeling conditions after deformation with the required accuracy for aECCI was not possible. However, enhanced BSE images were acquired and gave the necessary information for understanding the evolution of the microstructure already fully characterized before deformation.

EBSD gave (42 54 73)~(4 5 7) as surface plane so that seven channeling conditions or diffracting vectors **g** were accessible by tilting and rotating the specimen: $g_{1}=\;\left(1\;\bar{1}\;0\right)$, $g_{2}=\;\left(1\;1\;\bar{1}\right)$, $g_{3}=\;\left(3\;\bar{1}\;\bar{1}\right)$, $g_{4}=\;\left(3\;\bar{3}\;1\right)$, $g_{5}=\;\left(1\;3\;\bar{3}\right)$, $g_{6}=\;\left(1\;\bar{3}\;1\right)$, $g_{7}=\;\left(4\;0\;\bar{2}\right)$ (note that only the ECC image taken with ***g*_1_** is shown in Figure 2a). In such conditions, all defects are expected to be in contrast. Neither dislocation nor superdislocation are observed before deformation in Figure 2a. Only parallel linear contrasts (labelled NT) are clearly visible. In addition, they are aligned along the $\sim \left[2\;\bar{3}\;1\right]$ direction. Such BSE contrast is generally attributed to nano-twins (NTs) and is consistent with $\left[1\;1\;\bar{2}\right]\left(1\;1\;1\right)$ as a true twin system [20,21]. After deformation (see Figure 2b), no dislocation is visible, but clearly identifiable changes are localized in the vicinity of the indent (Area 1 and Area 2 in Figure 2b). In Area 1, near the imprint, a $\left[1\;1\;\bar{2}\right]\left(1\;1\;1\right)$ deformation NT was created. At the other side of the imprint (Area 2) NT5 extends along the $\sim \left[2\;\bar{3}\;1\right]$. Note that NT7 visible in Figure 2b comes from a neighbor imprint. 

### 3.2. Microstructure Evolution of ROI2

ROI2 is composed by two twinned grains A (left) and B (right) with their surface plane as (42 54 73)~(4 5 7) and (16 325 946)~(0 1 3), respectively (see Figure 3a). The common direction on the sample surface for both grains A and B was $\left[2\;\bar{3}\;1\right]$. The {1 1 1}-plane, which intercepts both the (4 5 7) plane and the (0 1 3) plane along $\left[2\;\bar{3}\;1\right]$ is the (1 1 1). Note also that an NT aligned along $\sim \left[2\;\bar{3}\;1\right]$ is visible (labelled NT8 in Figure 3) and consistent with $\left[1\;1\;\bar{2}\right]\left(1\;1\;1\right)$. The vertical dislocations (i.e., almost perpendicular to the sample surface) either isolated or stacked into a wall in grain A (Figure 3a) were analyzed by aECCI in order to determine their Burgers vectors. Using the diffracting conditions $g_{1}$ to $g_{7}$ previously mentioned with invisibility criteria led to $\pm \frac{1}{2}\left[1\;1\;0\right]$ as the Burgers vector. 

Unfortunately, good channeling conditions were not reachable in the right (0 1 3) grain, resulting in the non-characterization of the isolated vertical dislocations. 

Figure 3b and its schematic show ROI2 after deformation. The 500 µN indent was made in the (0 1 3) grain near the TB. Around this indent, two similar features (labelled B1 and B2 in Figure 3b) are observed. Parallel to the TB (i.e., in B1), a set of parallel dislocation traces is visible (yellow arrows in Figure 3b). They are localized in an elliptical area forming a buckling (B1) extending far away from the imprint in the $\left[2\;\bar{3}\;1\right]$ direction. Such buckling areas were already reported, but not explained for TiAl alloys [18,22].

In addition, an NT contrast (blue arrow in Figure 3b) is observed inside B1, and it is parallel to $\left[2\;\bar{3}\;1\right]$, consistent with the $\left[1\;1\;\bar{2}\right]\left(1\;1\;1\right)$ true twinning system.

Perpendicular to the TB (i.e., along $\left[\bar{5}\;\bar{3}\;1\right]$), another buckling area B2 is observed, and it could extend because it was blocked by the TB. In the neighbor (4 5 7) grain, no change is observed compared to the initial state, even if the TB is distorted locally where B2 was in contact. Outside both buckling areas, no other defect is observed. 

## 4. Discussion

From observations of the evolution of microstructures of ROI1, two assessments can be made:

1. At RT, twinning was observed to be the main deformation mechanism, in agreement with literature [2,7,8]. However, this runs contrary to Zambaldi et al., who prefer to suggest that ordinary dislocation glide is the main deformation mechanism at RT (without totally excluding twinning) from observations by atomic force microscopy around high-load (3000 µN) imprints [18].
2. Deformation was observed to be localized near the indent.

In many materials, buckling areas such as those characterized in ROI2 are associated with a canalization of the deformation, generally taking its origin from the accommodation of twins [23]. Although the accommodation of $\frac{1}{6}\langle 1\;1\;\bar{2}]\{1\;1\;1\}$ twin by $\frac{1}{2}\langle 1\;1\;0]\left\{1\;1\;1\right\}$ ordinary dislocations was already reported by TEM experiments in TiAl alloys [24,25], no mechanism was proposed.

From this knowledge, and taking into account our results, we propose the following scenario (see Figure 3c):

- Under the indent, the $\left[1\;1\;\bar{2}\right]\left(1\;1\;1\right)$ NT was formed.
- The stress concentration at the tip of the $\left[1\;1\;\bar{2}\right]\left(1\;1\;1\right)$ NT nucleated ordinary $\pm \frac{1}{2}\left[1\;1\;0\right]$ dislocation loops gliding in the $\left(1\;\bar{1}\;1\right)$ planes. The dislocation loops formed an ellipsoid surrounding the NT, thus producing lines after projection on the observation plane.
- The elliptical area or B1 grew by adding successive dislocation loops at its extremity.
- B1 extended until it met an obstacle, such as the TB (for B2 for example).
- At the location where B2 intercepts the TB, a stress concentration appeared. It resulted in a local distortion of the boundary. Therefore, the TB seems to be a strong obstacle to the propagation of the deformation, and at higher load it may cause microcracking at its vicinity, as observed in References [18,25,26].

Furthermore, we can suggest that the low load used (500 µN) was just high enough to generate a complex and non-uniaxial stress field at the tip of the indent. This led to the activation of the main deformation mechanism (i.e., twinning), but it was too low for dislocation glide. For higher loads, both mechanisms were activated subsequently, and led to the formation of a buckling area, according to the previous scenario. 

## 5. Conclusions

In summary, RT nanoindentation tests combined with aECCI observations before and after deformation brought novel insights into the γ-TiAl deformation mechanisms:

1. At RT, twinning was observed to be the main deformation mechanism.
2. Twinning was accommodated by ordinary dislocation mechanism, leading to the canalization of the deformation.
3. TB could play the role of obstacle to the propagation of deformation to neighbor grains, leading to a stress concentration at the vicinity of the boundary. Therefore, the true twin seems to be one of the weak links explaining the poor ductility of γ-TiAl at RT.
